# Clonal Integration and Root Morphological Plasticity of *Indocalamus latifolius* Under Heterogeneous Phosphorus Environments

**DOI:** 10.3390/plants15121857

**Published:** 2026-06-16

**Authors:** Bo Wang, Zhenya Yang

**Affiliations:** 1 Zhejiang Academy of Forestry, Hangzhou 310023, China; zjslxhbamboo@163.com; 2 Northwest Zhejiang Bamboo Forest Ecosystem Positioning Observation and Research Station, National Forestry and Grassland Administration, Hangzhou 310023, China

**Keywords:** phosphorus heterogeneity, clonal plant, clonal integration, root morphological plasticity, *Indocalamus latifolius*

## Abstract

Owing to clonal integration and morphological plasticity, clonal plants generally exhibit higher fitness in nutrient-heterogeneous environments. *Indocalamus latifolius* (Keng) McClure is a clonal plant with considerable economic value in China. However, the mechanisms of clonal integration and morphological plasticity in *I. latifolius* under phosphorus (P) heterogeneous conditions remain unclear. To clarify the mechanisms, a pot experiment was performed with *I. latifolius* clonal fragments consisting of mother ramet, rhizome, and daughter ramet. The experiment used a two-factor design with the following six treatments: P addition regime (uniform P addition, localized P addition to mother ramets, and localized P addition to daughter ramets) and rhizome status (connection vs. severance). Biomass allocation, root morphological plasticity, and the allocation pattern of P and non-structural carbohydrates were determined. The results showed that localized P addition increased the biomass of ramets growing in high-P patches and the total biomass of the clonal system compared with uniform P addition. Under three P supply regimes, rhizome connection significantly improved the biomass and P accumulation of daughter ramets relative to rhizome severance. In heterogeneous P environments, rhizome connection facilitated the proliferation of finer root and raised the soluble sugar concentrations in belowground tissues of ramets located in low-P patches compared with rhizome severance. In conclusion, resource allocation within the *I. latifolius* clonal system is prioritized toward daughter ramets and ramets in high-P patches. Clonal integration can promote compensatory root growth and morphological modification in ramets located in low-P patches. Localized P addition to mother ramets combined with the maintenance of rhizome connectivity between mother and daughter ramets is more conducive to the overall growth of the clonal system.

## 1. Introduction

Soil phosphorus (P) is typically distributed in a patchy pattern across spatial scales, representing soil P heterogeneity [1,2]. This nutrient distribution characteristic directly regulates plant growth, community structure, and ecosystem functioning. It is also a pivotal determinants of ecosystem productivity [3,4]. Plants can improve their adaptability to soil P heterogeneity through root morphological and architectural plasticity, enhanced internal P translocation capacity, or accelerated rhizosphere P activation [5,6]. Among them, clonal plants often exhibit higher fitness in heterogeneous nutrient environments by virtue of clonal integration and flexible morphological plasticity [7,8,9]. Therefore, investigating clarifies how clonal plants adapt to heterogeneous soil P. These results further establish theoretical guidelines for precise and P-efficient cultivation of clonal plants.

Clonal plant ramets often grow across soil patches with contrasting nutrient availability. They preferentially allocate propagules and nutrient uptake organs to nutrient-rich patches to display animal-like foraging behavior [10]. This morphological plasticity expands growing space under limited resources and improves nutrient acquisition from nutrient-rich patches. It also alleviates intra- and interspecific resource competition, sustaining high survival and growth performance of clonal plants [11,12]. Clonal integration represents another crucial adaptive mechanism for clonal plants. Interconnected ramets can translocate and share nutrients, water, photosynthates, and signaling substances via spacers such as rhizomes and stolons [13]. This process further regulates biomass allocation and morphological plasticity of individual ramets, thereby enabling a coordinated response to environmental heterogeneity [14,15,16]. For instance, under the mechanism of clonal integration, ramets located in nutrient-enriched patches usually assume a greater role in nutrient acquisition. Serving as a source of nutrient elements, they translocate nutrients to conspecific ramets growing in nutrient-poor soil patches. Such nutrient translocation lowers the risk of growth impairment caused by nutrient deficiency in these ramets [17].

Clonal integration exhibits distinct intensity and patterns, as it is regulated by multiple factors. These factors include the contrast of nutrient heterogeneity, the connection mode among ramets, inter-ramet distance, and the source–sink relationship among clonal ramets [18,19,20,21]. For instance, stoloniferous clonal plants generally exhibit a higher clonal integration intensity than rhizomatous species [22]. Clonal integration is generally intensified either under high-contrast heterogeneous nutrient patches or when source ramets occupy nutrient-rich microsites [23,24,25,26]. It is noteworthy that resource translocation via clonal integration and the construction of spacers entail definite metabolic costs. When these costs outweigh the benefits, resource integration and sharing among ramets will decline or even terminate [27,28]. Thus, the adaptive process of clonal plants to nutrient heterogeneity is highly complex and variable. Relevant inferences cannot be drawn based on the growth performance of other clonal species. Compared with nitrogen, light, water and other environmental factors, studies on clonal integration mechanisms of plants under P heterogeneity remain insufficient. Most existing researches focus on single distribution pattern of nutrient heterogeneity, ignoring the potential regulatory effect of source–sink relationships on clonal integration. Few studies combine clonal integration with morphological plasticity of core organs such as roots. These limitations hinder the understanding of the coupling mechanism between growth phenotype and internal resource allocation of clonal plants in heterogeneous P habitats.

As typical clonal plants, bamboos exhibit high growth and reproduction rates and strong nutrient foraging capacity [29]. Under subtropical low-P conditions, their adaptations to soil P heterogeneity shape population expansion toward adjacent forests and sustain long-term productivity. Therefore, this subject possesses distinctive research significance. Numerous studies have revealed obvious clonal integration and root morphological plasticity in bamboos under heterogeneous nutrient conditions. These phenotypic responses are modulated by source–sink relationships [30,31]. Such functional characteristics facilitate the rapid clonal expansion of bamboos toward adjacent forest stands [32,33]. *Indocalamus latifolius* (Keng) McClure belongs to the genus *Indocalamus* in the subfamily *Bambusoideae* of the family *Poaceae* [10]. Its leaves are widely used as wrapping materials for zongzi, a traditional delicacy of China’s Dragon Boat Festival. Accordingly, *I. latifolius* is a bamboo species with considerable economic and cultural values in China [34]. Furthermore, *I. latifolius* shows shade tolerance and can be intercropped under forests [35]. It possesses great regional ecological application potential in maintaining community stability, improving soil microenvironment and enhancing forest carbon sequestration capacity. *I. latifolius* is a typical rhizomatous clonal plant [36]. It presents obvious morphological plasticity and physiological integration under resource heterogeneity and environmental stress [33,35]. This species differs greatly from previously mentioned bamboos in rhizome type, spacer length and ramet size [37]. Various bamboo species have divergent foraging strategies and stress responses under heterogeneous nutrients [38]. Hence, *I. latifolius* may form unique clonal integration and morphological plasticity under heterogeneous P conditions. However, these mechanisms remain unclear at present.

Pot experiments were conducted using clonal fragments (mother ramet-rhizome-daughter ramet) of *I. latifolius.* Resource allocation patterns and root morphological plasticity were determined under contrasting P supply and rhizome connection treatments. To clarify the mechanisms underlying clonal integration and morphological plasticity of *I. latifolius* under heterogeneous P conditions, the following three hypotheses were proposed, as listed below: (1) the clonal integration of resources in *I. latifolius* is co-regulated by the spatial distribution pattern of P heterogeneity and the source–sink relationship among ramets; (2) compared with severed status, clonal integration under connected status promotes growth and P accumulation of ramets in low-P patches; and (3) under P heterogeneous conditions, *I. latifolius* exhibits specific root morphological plasticity, which is modulated by clonal integration. The findings provide theoretical references for precise fertilization, fertilizer reduction and stand nutrient management. They also help develop bamboo tending techniques adapted to heterogeneous low-P red soil.

## 2. Materials and Methods

### 2.1. Experimental Set up

The pot experiment was conducted in Xihu District, Hangzhou City, Zhejiang Province, China (120°01′ E, 30°13′ N). The experimental materials of *I. latifolius* were collected from the Bamboo Botanical Garden of Zhejiang Academy of Forestry Sciences, Hangzhou, China. In March, two-year-old mother plants of *I. latifolius* were excavated as entire individuals with undamaged rhizomes retained. Along the extending direction of the rhizome, 2 daughter ramets were retained within an area approximately 20 cm away from the mother plant. The combined unit consisting of the mother ramet, interconnecting spacer (rhizome), and daughter ramet was designated as one clonal system. Rectangular plastic pots (Dalian Iris Ohyama Trade & Industry Co., Ltd., Dalian, China) were adopted for cultivation. Each pot measured 40 cm long, 25 cm wide and 25 cm high. Each pot was divided into two equal parts by a 2 mm-thick plastic partition placed in the middle position. A 2 cm wide and 3 cm deep groove was cut into the upper part of the plastic partition. It permitted spacer growth through the partition and preserved physiological linkage between mother and daughter ramets. The experimental soil was an infertile yellow soil collected from the natural *I. latifolius* forest stand at Anji County, Huzhou City, Zhejiang Province. The soil was naturally air-dried and then passed through a 2 mm sieve. The soil organic matter was 30.2 g·kg^−1^. The total nitrogen, total P, and total potassium were 1.48 g·kg^−1^, 0.47 g·kg^−1^, and 11.4 g·kg^−1^ respectively. The hydrolyzable nitrogen, available P, and available potassium were 114.3 mg·kg^−1^, 7.64 mg·kg^−1^, and 123.5 mg·kg^−1^ respectively. The soil pH value was 5.02.

**Figure 1:**
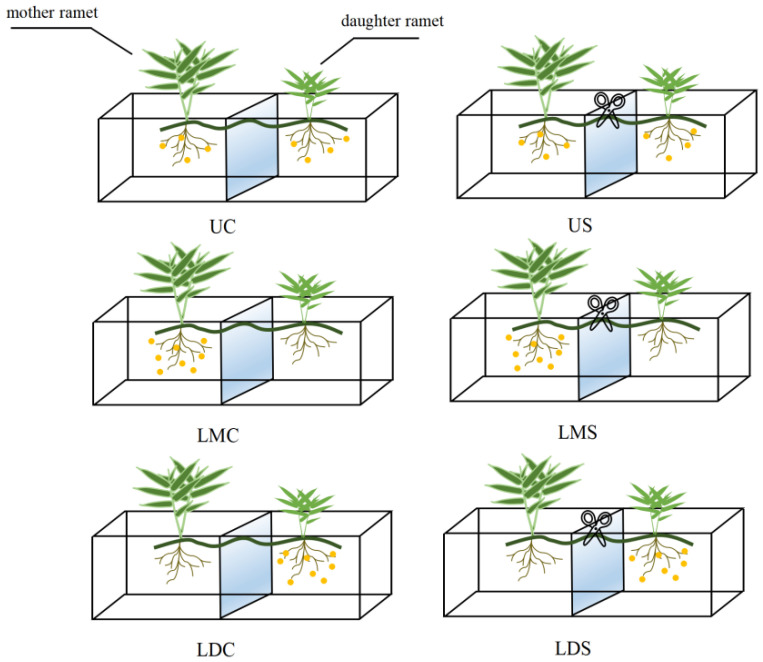
Schematic drawing of experimental treatments. Note: green lines: spacers; yellow dots: phosphorus fertilizer.

Thirty clonal systems with consistent growth performance were selected. They were randomly allocated into five independent blocks. Every block held six samples for six treatments. Each block served as one biological replicate. The six treatments were uniform P addition with spacers connected (UC), uniform P addition with spacers severed (US), localized P addition to mother ramets with spacers connected (LMC), localized P addition to mother ramets with spacers severed (LMS), localized P addition to daughter ramets with spacers connected (LDC), and localized P addition to daughter ramets with spacers severed (LDS) respectively (Figure 1). All plastic pots in each block were arranged randomly. They were repositioned every month during the experiment. This practice minimized the impacts of spatial differences in light, temperature and humidity.

P was supplied in the form of monocalcium phosphate monohydrate (Ca(H_2_PO_4_)_2_·CaSO_4_·H_2_O). Its available P content was 14.5% calculated as P_2_O_5_. The total P application amount per pot remained identical across all treatments. In treatments UC and US, the P application level was 200 mg·kg^−1^ soil. For LMC and LMS, P was added only to the mother ramet side. For LDC and LDS treatments, P was added only to the daughter ramet side. The mixed soil was placed into pots, and seedlings were transplanted afterwards. Before transplantation, the mother ramets were pruned to retain only 3–4 leaves and a small number of fine roots. One clonal system was transplanted into each pot. All pots were irrigated gravimetrically to keep soil moisture at 85% field capacity. For the spacer-severed treatments, the connecting spacer was cut with scissors at the plastic partition at the beginning of the experiment. Furthermore, spacers were inspected monthly to ensure disconnected spacers would not reconnect and new rhizomes could not cross the plastic partition. The pot experiment was carried out in a greenhouse. Ambient temperature ranged from 20 to 35 °C throughout cultivation. Light intensity was fixed at 80% natural sunlight with air humidity maintained at 75%.

### 2.2. Harvest and Measurements

The growth period of the pot experiment lasted 20 months. All 30 samples were harvested in November of the subsequent year, with 5 samples per treatment set as five replicates. Plant materials were separated into leaves, stems, rhizomes, and roots. All samples were transported to the laboratory in an incubator maintained at 0–2 °C. Root samples were rinsed and subsequently scanned using a dual-sided scanner (Regent Instruments Inc., Québec, QC, Canada). Root morphological parameters were analyzed using WinRhizo software (version 2.0). Measured indexes including root length, root surface area, average root diameter, and root length for the following five diameter grades: 0–0.5 mm, 0.5–1 mm, 1–1.5 mm, 1.5–2 mm, and >2 mm. The root length ratio of each diameter grade was calculated as the root length of the corresponding diameter grade divided by the total root length. Specific root length was calculated as the ratio of total root length to root dry mass. Leaves, stems, rhizomes, and roots were subjected to deactivation at 105 °C for 30 min, and subsequently oven-dried to constant weight at 65 °C. The dry mass of each plant organ was determined to represent biomass. All dried samples were fully ground and digested using the hydrogen peroxide–sulfuric acid digestion method. P content was then measured via the vanadomolybdate yellow colorimetric method [39]. The anthrone colorimetric method was adopted to determine the content of non-structural carbohydrates, including soluble sugar and starch [40].

### 2.3. Statistical Analysis

Differences in all measured traits across treatments were evaluated with SPSS 20.0 (SPSS Inc., Chicago, IL, USA). Before performing ANOVA procedures, the Shapiro–Wilk and Levene’s tests were adopted to assess the normal distribution and variance homogeneity of the experimental data. One-way analysis of variance was applied to compare differences among six treatments. Significance level was set at α = 0.05. The significance threshold for statistical difference was defined as *p* < 0.05. Fisher’s least significant difference (LSD) post hoc tests were used to identify significant differences among treatment levels. The main reasons for selecting LSD are as follows: uniform sample replication was achieved across all six experimental treatments, satisfying the applicable assumptions of LSD. This condition guarantees reliable comparison outcomes. LSD possesses superior test sensitivity to distinguish inherent inter-treatment discrepancies. This test is widely used for pairwise comparisons in forestry trials with balanced sample sizes similar to our experiment. All graphs were generated by Origin 9.0 software.

## 3. Results

### 3.1. Effects of Different Treatments on Biomass Allocation

**Figure 2:**
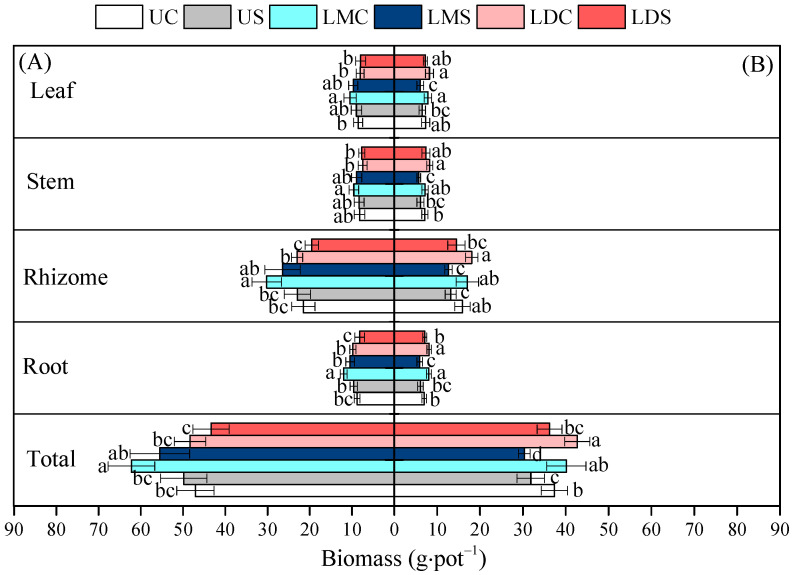
Biomass allocation of *Indocalamus latifolius* under different treatments. Different letters indicate significant differences among different treatments (*p* < 0.05). LSD test was used for post hoc analysis. The values represent means ± SE. Sample size: *n* = 5. (**A**): mother ramet; (**B**): daughter ramet. Six treatments: (UC, US): uniform phosphorus addition (spacers connected/severed); (LMC, LMS): localized phosphorus addition to mother ramets (spacers connected/severed); (LDC, LDS): localized phosphorus addition to daughter ramets (spacers connected/severed).

The connection status (connected or severed) between mother and daughter ramets exerts a significant effect on biomass accumulation of mother and daughter ramets. Specifically, under uniform P addition, the connected treatment (UC) significantly increased rhizome biomass and total biomass of daughter ramets in comparison with the severed treatment (US) (*p* < 0.05). Under localized P addition to mother ramets, the connected treatment (LMC) significantly increased the root biomass of mother ramets, as well as the leaf, stem, rhizome, and root biomass and total biomass of daughter ramets (*p* < 0.05), in comparison with the severed treatment (LMS). By contrast, no significant differences were observed in the biomass of other organs and the total biomass of mother ramets between the two treatments. Under localized P addition to daughter ramets, the connected treatment (LDC) significantly enhanced the rhizome and root biomass of both mother and daughter ramets, along with the total biomass of daughter ramets (*p* < 0.05), relative to the severed treatment (LDS) (Figure 2).

Under connected conditions, relative to the uniform P addition treatment (UC), localized P addition to mother ramets (LMC) significantly increased root biomass of daughter ramets, as well as root biomass, rhizome biomass, leaf biomass, and total biomass of mother ramets. Localized P addition to daughter ramets (LDC) significantly increased root biomass, stem biomass, and total biomass of daughter ramets, while exhibiting no significant effects on the biomass of mother ramets (Figure 2).

The total biomass of the clonal system peaked under the LMC treatment, being significantly higher than that of all other treatments. Compared with severed treatments, the connected treatments increased total biomass by 3.3% (UC vs. US), 19.2% (LMC vs. LMS), and 14.4% (LDC vs. LDS), respectively. Under connected conditions, compared with uniform P addition (UC), localized P addition to mother ramets (LMC) increased the biomass of mother ramets, daughter ramets, and the whole clonal system by 32.2%, 7.4% and 21.2%, respectively. Similarly, localized P addition to daughter ramets (LDC) increased the corresponding biomass by 2.7%, 14.3%, and 7.8%, respectively. These findings demonstrate that clonal integration consistently benefits biomass accumulation of the entire clonal system across all P distribution patterns. Furthermore, the positive effect can be greatly strengthened when mother ramets are located in high-P patches (Figure 2).

### 3.2. Effects of Different Treatments on Root Morphology

**Figure 3:**
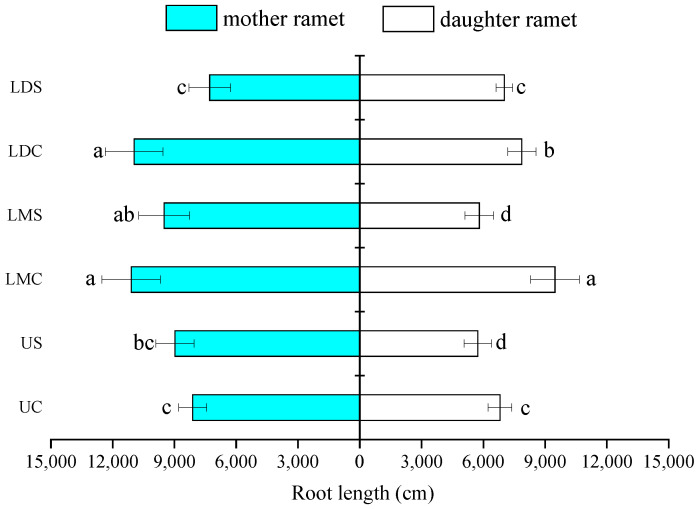
Root length of *Indocalamus latifolius* under different treatments. Different letters indicate significant differences among different treatments (*p* < 0.05). LSD test was used for post hoc analysis. The values represent means ± SE. Sample size: *n* = 5.

UC exhibited a significantly higher root length of daughter ramets than US, with an increase of 18.9%. These findings demonstrated that clonal integration under homogeneous P conditions tended to allocate greater resources to daughter ramets, thereby stimulating their root development. Under heterogeneous P supply, clonal integration significantly facilitated root growth of ramets in low-P patches. Specifically, compared with the LMS treatment, the LMC treatment significantly enhanced the root length of daughter ramets by 63.3%. In comparison with the LDS treatment, the LDC treatment increased the root length of mother and daughter ramets by 50.4% and 12.1%, respectively (*p* < 0.05). Relative to homogeneous P environments, *I. latifolius* possessed a stronger physiological integration capacity under heterogeneous P conditions. Such integration promoted root proliferation of ramets in low-P patches to compensate for limited soil P availability (Figure 3). Furthermore, under connected conditions, both localized P addition treatments significantly increased the root length of mother and daughter ramets in comparison with uniform P addition.

**Figure 4:**
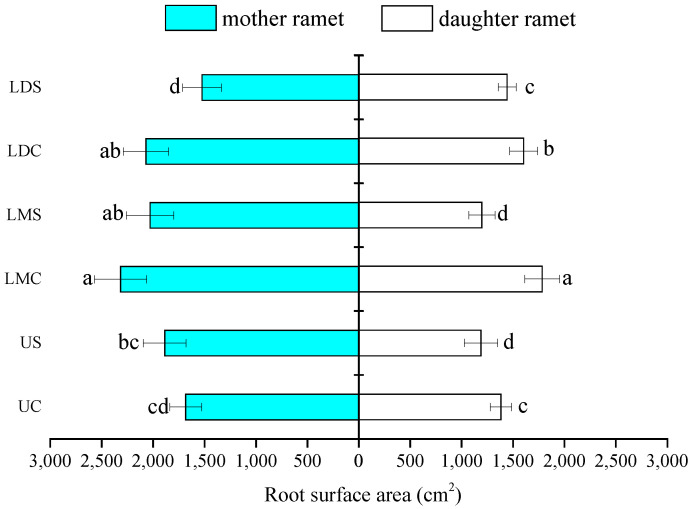
Root surface area of *Indocalamus latifolius* under different treatments. Note: different letters indicate significant differences among different treatments (*p* < 0.05). LSD test was used for post hoc analysis. The values represent means ± SE. Sample size: *n* = 5.

Consistent with the root length results, under homogeneous P addition, the connected treatment (UC) significantly increased the root surface area of daughter ramets by 16.2% relative to the severed treatment (US). Compared with the LMS treatment, the LMC treatment significantly increased the root surface area of daughter ramets by 49.0%. In comparison with the LDS treatment, the LDC treatment significantly enhanced the root surface area of mother and daughter ramets by 35.7% and 11.1%, respectively (Figure 4).

**Figure 5:**
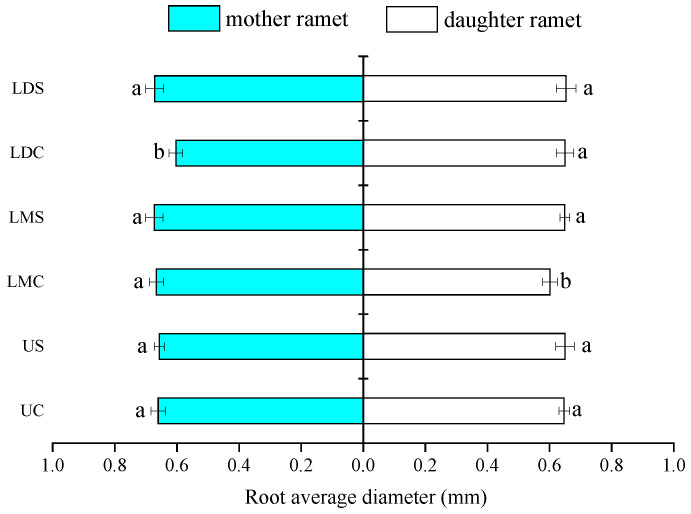
Average root diameter of *Indocalamus latifolius* under different treatments. Note: different letters indicate significant differences among different treatments (*p* < 0.05). LSD test was used for post hoc analysis. The values represent means ± SE. Sample size: *n* = 5.

**Figure 6:**
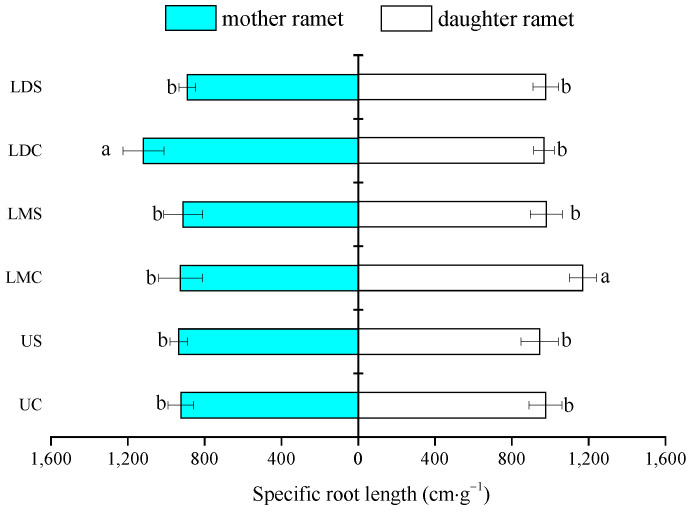
Specific root length of *Indocalamus latifolius* under different treatments. Different letters indicate significant differences among different treatments (*p* < 0.05). LSD test was used for post hoc analysis. The values represent means ± SE. Sample size: *n* = 5.

**Figure 7:**
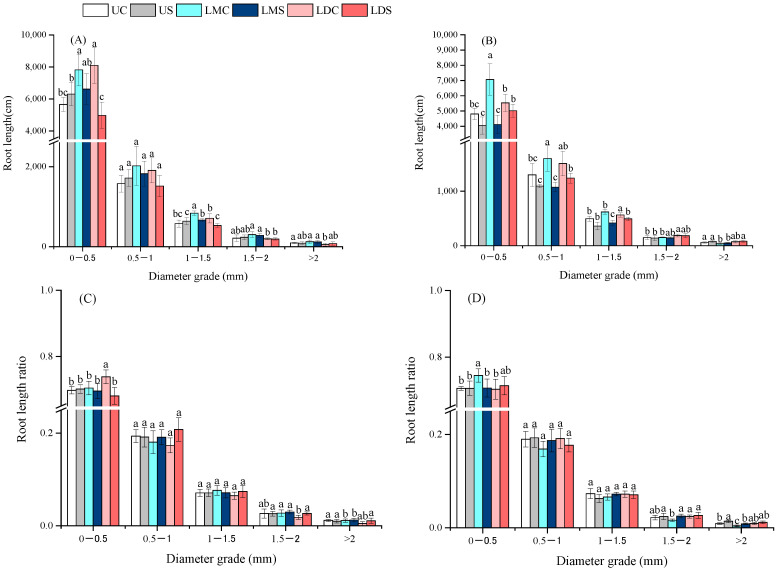
Root length and length ratio across diameter grades under different treatments. (**A**,**B**): root length of mother and daughter ramets; (**C**,**D**): root length ratio of mother and daughter ramets. Different letters indicate significant differences among different treatments (*p* < 0.05). LSD test was used for post hoc analysis. The values represent means ± SE. Sample size: *n* = 5.

Notably, clonal integration facilitates compensatory root growth and induces morphological modifications of ramets in low-P microhabitats under localized P heterogeneity. Specifically, under the ramet-connected conditions, both LMC and LDC significantly reduced the average root diameter and increased the specific root length of ramets within low-P patches relative to the UC treatment (Figure 5 and Figure 6). Such regulatory responses were not observed under the ramet-severed condition. Compared with the LMS treatment, LDC reduced the average root diameter and increased the specific root length of daughter ramets. Similarly, relative to the LDS treatment, LDC decreased the average root diameter and enhanced the specific root length of mother ramets. The proliferation of a large number of fine roots in ramets growing in low-P patches driven by clonal integration constitutes the primary mechanism underlying the reduced average root diameter and increased specific root length. In detail, compared with the LMS treatment, LMC significantly increased the root length and root length ratio in the 0–0.5 mm diameter grade of daughter ramets, and reduced the root length ratio in both the 1.5–2 mm and >2 mm diameter grades of daughter ramets. Relative to the LDS treatment, LDC significantly enhanced the root length and root length ratio in the 0–0.5 mm diameter grade of mother ramets, while decreasing the root length ratio in the 1.5–2 mm diameter grade of mother ramets (Figure 7).

### 3.3. Effects of Different Treatments on the Phosphorus Allocation of Indocalamus latifolius

Compared with the US treatment, UC significantly increased the P content in the leaves of daughter ramets. Apart from this, there were no significant differences in the P content of other organs in both mother and daughter ramets between US and UC. Under localized P addition to mother ramets, LMC significantly reduced P content in the rhizomes and stems of mother ramets, while increasing P content in the leaves and roots of daughter ramets relative to LMS. Under localized P addition to daughter ramets, LDC significantly increased P content in the roots and leaves of mother ramets compared with LDS (Table 1).

Under uniform P addition, connection and severance exerted no significant effects on the P accumulation of individual organs and the total P accumulation of mother ramets. Compared with the US treatment, UC significantly increased the P accumulation in leaves and stems of daughter ramets, as well as the total P accumulation of daughter ramets (Table 2).

Compared with LMS, LMC had no significant effect on the P accumulation in various organs of mother ramets, but significantly increased the P accumulation in leaves, stems, roots, and rhizomes, as well as the total P accumulation of daughter ramets, with respective increases of 89.6%, 39.4%, 71.1%, 47.9%, and 58.6%. Compared with LDS, LDC significantly increased the P accumulation in leaves and roots, as well as the total P accumulation of mother ramets, with respective increases of 47.1%, 56.8% and 31.7%. Compared with the ramet severance treatment, the total P accumulation of the clonal system in the connection treatment increased by 3.3% (UC vs. US), 11.2% (LMC vs. LMS), and 13.9% (LDC vs. LDS), respectively. Compared with the UC treatment, LMC significantly increased the P accumulation of mother ramets by 84.5% and the total P accumulation of the clonal system by 44.6%, while LDC significantly increased the P accumulation of daughter ramets by 46.2% and the total P accumulation of the clonal system by 24.7% (Table 2).

### 3.4. Effects of Different Treatments on the Non-Structural Carbohydrate Allocation of Indocalamus latifolius

Under uniform P addition, no significant differences were observed in starch content of roots, rhizomes, stems, and leaves for both mother and daughter ramets between the connected and severed spacer treatments. Under localized P addition to mother ramets, LMC significantly decreased the starch content in the roots of mother ramets, as well as in the stems and roots of daughter ramets, relative to LMS. Under localized P addition to daughter ramets, LDC significantly reduced starch content in stems, roots, and rhizomes of mother ramets, as well as in stems of daughter ramets, relative to LDS (Table 3). These findings indicate that clonal integration facilitates the growth of ramets in low-P microhabitats, accompanied by substantial starch consumption. Compared with the UC treatment, the LMC treatment significantly decreased the starch content in the roots of daughter ramets, while the LDC treatment significantly reduced the starch content in stems and roots of mother ramets.

Under uniform P addition, UC significantly increased the soluble sugar content in rhizomes and roots of daughter ramets relative to US. Under localized P addition to mother ramets, compared with LMS, LMC significantly decreased the soluble sugar content in leaves and stems of mother ramets, while increasing that in roots and rhizomes of daughter ramets. Under localized P addition to daughter ramets, LDC significantly increased the soluble sugar content in roots and rhizomes of mother ramets, as well as in roots of daughter ramets, in comparison with LDS (Table 4).

## 4. Discussion

### 4.1. Biomass Allocation Strategies of Clonal Systems

Under identical rhizome connection status, growth discrepancies between localized and uniform P addition were compared to determine the regulatory effects of P heterogeneity on *I. latifolius*. In this study, soil P heterogeneity was more conducive to total biomass accumulation of the *I. latifolius* clonal system than homogeneous P conditions. This phenomenon is widely observed among clonal plants. For example, ten clonal plant species have been documented to exhibit significantly better growth performance in nutrient-heterogeneous soils than in homogeneous soils [41]. Additionally, under localized P supply, the clonal system of *I. latifolius* tended to allocate greater biomass to ramets located in high-P patches. Consistently, a study on *Alternanthera philoxeroides* (Mart.) Griseb. reported that localized nutrient addition did not alter the overall biomass of the whole plant but significantly increased the biomass proportion of ramets within high-nutrient patches [17].

With identical P application regimes, growth differences between connected and severed rhizome treatments were compared to evaluate the regulatory role of clonal integration in *I. latifolius*. In the present study, clonal integration mechanism of *I. latifolius* is regulated by source–sink relationships, following an allocation principle prioritizing daughter ramets. Consequently, clonal integration significantly enhances daughter ramet growth under all three P addition treatments. By contrast, previous studies on other clonal plants have reported that clonal integration is nearly absent under low nutrient patch contrast or homogeneous nutrient conditions [19,42]. Such differences may be related to ramet size and clonal connection distance. For instance, the maximum distance of clonal integration for nitrogen in the *Phyllostachys glauca* McClure clonal system is 2.82 m [31]. Studies on *Indocalamus decorus* Q. H. Dai have also shown that shorter spacer length corresponds to greater clonal integration intensity [33]. Compared with uniform P addition, maintaining spacer connection under P heterogeneity produced a larger improvement in the biomass of the clonal system. These findings support the previous conclusion that the ecological benefits derived from clonal integration increase correspondingly with rising nutrient patch contrast [43].

In the present study, clonal integration among *I. latifolius* ramets is bidirectional and not entirely determined by source–sink relationships. When daughter ramets grow in high-P patches, clonal integration still facilitates the growth of mother ramets in low-P patches. However, such beneficial effect on low-P ramets is markedly lower than the effect when mother ramets are located in high-P patches. This may be because the driving force of P heterogeneity, which promotes resource translocation from ramets in high-P patches to those in low-P patches, conflicts with the source-sink gradient that drives resource transport from mother ramets to daughter ramets. Such conflict weakens the growth-promoting effect of clonal integration on ramets in low-P patches. In contrast, when the two driving forces act in the same direction, the facilitating effect of clonal integration on plant growth can be greatly enhanced. Consequently, the total biomass of the clonal system under LDC was significantly lower than that under LMC. The regulatory mechanism of bidirectional integration cannot be clarified in this study. Further transcriptomic, proteomic, hormonal and enzymatic activity analyses focusing on P transporters including PHT1 family and PHO1, as well as sucrose synthase, will help reveal the underlying mechanism. In addition, *I. latifolius* prioritizes resource investment in P-foraging organs when supplies of P, carbon and other resources from high-P patches are insufficient. Similarly, *Phyllostachys edulis* (Carrière) J. preferentially allocates biomass to roots and increases the root-shoot ratio when low soil P constrains carbon assimilation [44]. Notably, under heterogeneous P conditions, clonal integration also induced compensatory growth of P foraging organs in ramets within high-P patches. This represents a typical environmentally induced clonal division of labor, in which functional organs of ramets in high-resource patches exhibit compensatory growth to undertake greater resource acquisition tasks [45,46].

In conclusion, biomass allocation of the *I. latifolius* clonal system is jointly regulated by P distribution patterns and source–sink relationships. The clonal system preferentially allocates biomass to high-P patches and daughter ramets. Under P heterogeneity, clonal integration can facilitate the growth of ramets in low-P patches and induce compensatory growth of P foraging organs in high-P patches. Such resource allocation patterns reflect the intrinsic adaptive mechanism enabling *I. latifolius* to sustain stable populations under heterogeneous P conditions. These findings also provide theoretical support for population conservation and precise nutrient regulation of bamboos in natural communities. Furthermore, against the ecological backdrop of bamboo encroachment into adjacent forest stands, our results provide a novel perspective for exploring the mechanisms underlying bamboo population expansion in heterogeneous P habitats.

### 4.2. Root Morphological Plasticity of Clonal Systems

Both clonal integration and heterogeneous P addition significantly promoted root growth of the *I. latifolius* clonal system. Similar findings have also been reported in *P. edulis*, where localized nitrogen and P addition significantly enhanced the growth of underground rhizome–root systems [8,24]. Nevertheless, the phenomenon of compensatory root growth in low-P patches mediated by clonal integration observed in this study is relatively rare among clonal plants. Previous studies have confirmed that low soil P can induce compensatory growth and morphological modification of fine roots in some non-clonal plants [47]. However, no low P-induced compensatory root growth has been observed in previous studies on bamboo species under either uniform low P or heterogeneous P conditions [44,48]. Instead, bamboo plants tend to allocate more roots to high-P regions [8]. Notably, under localized P addition, the compensatory root growth in low-P regions was accompanied by shifts in root morphology and resource investment strategies. That is, an increase in the ratio of fine roots and a reduction in the ratio of coarse roots ultimately reduced average root diameter and increased specific root length. This may reflect a cost–benefit trade-off mechanism adopted by *I. latifolius* during clonal integration under heterogeneous nutrient conditions. Compared with coarse roots, fine roots present higher nutrient absorption efficiency and lower construction cost. This trait is particularly critical for plant growth and survival under nutrient-limited and carbon-deficient conditions [49]. The clonal system of *I. latifolius* tends to proliferate a large number of low-cost roots in low-P patches to improve P foraging efficiency, thereby minimizing the investment risk in low-resource regions. Finer roots are generally distributed at the terminal parts of root branching systems, corresponding to lower root orders in functional classification, and possess stronger P absorption capacity [50]. Roots of the first to third orders primarily undertake nutrient uptake. By contrast, thicker fourth and fifth order roots exhibit reduced cortical thickness and mycorrhizal colonization rate, with weakened nutrient absorption ability, and mainly function in nutrient translocation [51]. Low P levels can reduce root diameter and increase specific root length. Such morphological changes enable roots to explore larger soil volume and enhance total P uptake of plants [52]. Similarly, the increase in the ratio of fine roots induced by soil P deficiency has also been documented in studies on *P. edulis* [53].

### 4.3. Phosphorus and Photosynthate Allocation of Clonal Systems

In this study, *I. latifolius* transported more P to daughter ramets under homogeneous P conditions. By contrast, more P was allocated to ramets in high-P patches under heterogeneous P environments. Such a P allocation strategy represents a widespread risk-spreading tactic in clonal plants, which distributes ecological risk through targeted resource storage [10,54]. In this study, localized P addition increased the P content and P accumulation of ramets in high-P patches, while clonal integration benefited P accumulation of ramets in low-P patches. It can be inferred that localized P application enhances the clonal integration capacity of *I. latifolius.* This clonal integration mechanism enables P translocation from ramets in high-P patches to low-P patches, compensating for nutrient deficit and sustaining basic metabolic growth. Nevertheless, P isotope labeling is required for quantitative analysis to fully clarify the P transport mechanism. In this study, when P translocation from sources to sinks or from high-P patches to low-P patches is insufficient, clonal integration preferentially allocates P to leaves and roots. Nevertheless, allocation strategies differ among nutrient elements. For instance, under nitrogen heterogeneity, nitrogen translocated from mother to daughter ramets of *P. edulis* is preferentially allocated to roots and rhizomes rather than leaves [24]. Therefore, P clonal integration of *I. latifolius* is bidirectional under heterogeneous P conditions and is regulated by source–sink relationships, following the allocation principle of priority to high-P patches and daughter ramets.

Compared with uniform P addition, the effects of clonal integration on the contents of starch and soluble sugar were significantly enhanced under heterogeneous P conditions. Rapid organ growth in *I. latifolius* is generally accompanied by reduced starch content. This may be attributed to the substantial consumption of stored carbohydrates to support compensatory root growth mediated by clonal integration. Previous studies have indicated that low P constrains starch transformation in leaves, thereby promoting starch accumulation, which may also explain the relatively stable leaf starch content of ramets in low-P patches of *I. latifolius* [55]. Overall, organs with growth promoted by clonal integration were accompanied by increases in soluble sugar content. Previous studies have confirmed that plants can convert a large amount of stored starch into soluble sugars to supply substances and energy for rapid growth [56]. Clonal integration elevates soluble sugar levels in roots and rhizomes of daughter ramets growing in low-P patches while reducing soluble sugar concentrations in mother ramets located in high-P patches. The increased soluble sugar in daughter ramets is most likely derived from translocation via clonal integration from high-P patches, acting to support local growth. In addition, when carbon supply is insufficient under low P conditions, roots, as organs with P absorption function, have a higher priority. Similarly, previous studies have found that under heterogeneous nutrient conditions, carbon from ramets in high-nutrient patches is mainly allocated to the roots of daughter ramets [25].

For the management of *I. latifolius*, localized fertilization around mother ramets is proposed to replace conventional uniform fertilization, so as to enhance P-use efficiency. Rhizome damage should be prevented during soil cultivation and weeding to sustain the linkage between mother and daughter ramets. Under current simple clonal system and controlled experimental conditions, localized P addition on mother ramets yielded optimal growth performance. Nevertheless, further research and field practice are needed to establish fertilization strategies for *I. latifolius* forests in natural environments.

This study has inherent limitations. Pot cultivation of *I. latifolius* fails to perfectly mimic growth conditions in farmland and natural bamboo stands. Wild populations possess intricate underground rhizome systems composed of interconnected ramets at diverse ages. Moreover, field environments exhibit heterogeneous availability of nitrogen, light and water. Such environmental variations may induce more complex morphological plasticity and clonal integration responses in this bamboo species. In addition, leaves, stems and rhizomes of bamboo develop asynchronously. No sequential growth data were obtained during the 20-month experimental period, which limits the exploration of growth dynamics under P heterogeneity. Further studies will focus on comprehensive assessments of growth traits at different developmental stages under complex field conditions. This study explored clonal integration and resource translocation mechanisms yet failed to directly track P and carbon transport among ramets. Isotope tracing technology will provide more solid evidence for clarifying clonal integration mechanisms in future research.

## 5. Conclusions

In conclusion, the present findings fully confirm our three initial hypotheses. First, consistent with Hypothesis 1, resource allocation within the *I. latifolius* clonal system is co-regulated by P heterogeneity and source–sink relationships. Resources are preferentially allocated to daughter ramets and ramets located in high-P patches. This pattern represents adaptive strategies of clonal plants under uneven nutrient distribution. Second, under localized P application, clonal integration promotes the accumulation of biomass, P, and soluble sugars in ramets within low-P patches, verifying Hypothesis 2. Third, under localized P application, clonal integration induces compensatory proliferation of finer roots in low-P ramets, supporting Hypothesis 3. In the context of bamboo population ecology, this clonal integration and root morphological plasticity clarifies the intrinsic mechanism whereby *I. latifolius* sustains population persistence under P-heterogeneous conditions. It provides theoretical references for population conservation and rational nutrient management of bamboos in natural habitats. On a broader ecosystem scale, these findings offer new perspectives for exploring population expansion mechanisms of bamboos in low-P and P-heterogeneous habitats. Localized P application to mother ramets while retaining the connections between mother and daughter ramets optimizes the growth of simple clonal systems of *I. latifolius.* However, these conclusions are only valid under the present experimental conditions and cannot be adopted as general fertilization guidelines for field cultivation prior to verification via field trials.

## Figures and Tables

**Table 1:** Phosphorus content of *Indocalamus latifolius* under different treatments.

Ramets	Treatments	P Content (mg·g^−1^)			
		Leaves	Stems	Roots	Rhizome
Mother ramets	UC	1.67 ± 0.11 b	1.30 ± 0.09 bc	1.59 ± 0.14 bc	1.48 ± 0.11 c
	US	1.74 ± 0.13 b	1.43 ± 0.10 b	1.65 ± 0.07 b	1.55 ± 0.06 c
	LMC	3.26 ± 0.13 a	1.59 ± 0.14 b	2.35 ± 0.11 a	1.90 ± 0.12 b
	LMS	3.42 ± 0.25 a	2.03 ± 0.14 a	2.56 ± 0.18 a	2.22 ± 0.16 a
	LDC	1.40 ± 0.06 c	1.22 ± 0.11 c	1.77 ± 0.10 b	1.44 ± 0.10 c
	LDS	1.02 ± 0.07 d	1.17 ± 0.09 c	1.37 ± 0.08 c	1.39 ± 0.08 c
Daughter ramets	UC	1.82 ± 0.12 b	1.58 ± 0.09 b	1.65 ± 0.13 b	1.53 ± 0.07 b
	US	1.55 ± 0.09 c	1.48 ± 0.11 bc	1.59 ± 0.07 b	1.54 ± 0.12 b
	LMC	1.63 ± 0.14 bc	1.33 ± 0.07 c	1.61 ± 0.10 b	1.43 ± 0.14 bc
	LMS	1.11 ± 0.07 d	1.21 ± 0.13 c	1.28 ± 0.09 c	1.26 ± 0.11 c
	LDC	2.99 ± 0.20 a	1.93 ± 0.15 a	2.45 ± 0.24 a	2.07 ± 0.13 a
	LDS	3.02 ± 0.17 a	2.02 ± 0.17 a	2.40 ± 0.11 a	2.21 ± 0.11 a

Note: different letters indicate significant differences among different treatments (*p* < 0.05). LSD test was used for post hoc analysis. The values represent means ± SE. Sample size: *n* = 5.

**Table 2:** Phosphorus accumulation of *Indocalamus latifolius* under different treatments.

Ramets	Treatments	P Accumulation (mg·Pot^−1^)				
		Leaves	Stems	Roots	Rhizome	Total
Mother ramets	UC	14.24 ± 1.67 b	10.65 ± 1.59 cd	14.31 ± 1.37 cd	33.33 ± 5.25 b	72.53 ± 6.25 b
	US	15.63 ± 2.58 b	11.86 ± 1.58 bc	16.17 ± 1.71 bc	35.51 ± 5.65 b	79.18 ± 10.47 b
	LMC	34.16 ± 4.85 a	15.19 ± 1.93 ab	28.17 ± 1.95 a	56.28 ± 4.81 a	133.8 ± 9.93 a
	LMS	33.03 ± 2.35 a	18.06 ± 2.77 a	26.69 ± 2.94 a	58.26 ± 8.13 a	136.04 ± 13.74 a
	LDC	12.04 ± 1.62 b	9.94 ± 1.28 cd	18.15 ± 2.03 b	37.36 ± 4.67 b	77.5 ± 8.47 b
	LDS	8.19 ± 1.33 c	9.00 ± 1.08 d	11.58 ± 1.96 d	30.08 ± 3.02 b	58.84 ± 6.93 c
Daughter ramets	UC	13.36 ± 2.07 b	11.22 ± 1.05 b	11.60 ± 1.74 bc	24.08 ± 2.95 bc	60.26 ± 6.58 b
	US	10.12 ± 1.26 c	9.01 ± 1.43 c	9.86 ± 1.17 c	20.42 ± 2.12 c	49.40 ± 5.38 c
	LMC	12.71 ± 1.59 bc	9.58 ± 1.28 bc	12.72 ± 1.26 b	23.27 ± 4.27 bc	58.27 ± 8.08 bc
	LMS	6.70 ± 0.84 d	6.87 ± 0.30 d	7.43 ± 0.62 d	15.73 ± 1.13 d	36.74 ± 2.11 d
	LDC	22.51 ± 3.12 a	14.70 ± 2.2 a	19.70 ± 2.44 a	31.22 ± 3.67 ab	88.13 ± 10.26 a
	LDS	21.93 ± 2.15 a	14.90 ± 2.25 a	17.03 ± 1.48 a	32.76 ± 4.83 a	86.62 ± 8.46 a

Note: different letters indicate significant differences among different treatments (*p* < 0.05). LSD test was used for post hoc analysis. The values represent means ± SE. Sample size: *n* = 5.

**Table 3:** Starch content of *Indocalamus latifolius* under different treatments.

Ramets	Treatments	Starch Content (%)			
		Leaves	Stems	Roots	Rhizome
Mother ramets	UC	12.54 ± 1.06 ab	17.96 ± 0.87 a	22.35 ± 1.68 ab	33.56 ± 2.56 ab
	US	13.56 ± 0.78 a	17.65 ± 1.00 a	23.49 ± 1.76 ab	32.72 ± 1.97 ab
	LMC	11.98 ± 0.99 ab	17.14 ± 1.07 ab	21.97 ± 0.78 b	33.00 ± 2.70 ab
	LMS	13.52 ± 0.88 a	18.62 ± 1.38 a	24.56 ± 0.90 a	34.93 ± 1.84 a
	LDC	11.47 ± 1.07 b	15.54 ± 0.57 b	18.17 ± 1.17 c	30.93 ± 0.89 b
	LDS	12.80 ± 0.61 ab	17.99 ± 1.08 a	22.77 ± 1.44 ab	33.80 ± 1.38 a
Daughter ramets	UC	11.48 ± 0.86 ab	15.64 ± 1.08 bc	20.50 ± 0.85 a	29.26 ± 2.21 a
	US	12.65 ± 0.89 a	16.22 ± 1.00 ab	21.50 ± 1.62 a	31.01 ± 1.95 a
	LMC	10.82 ± 0.79 b	14.02 ± 0.77 c	17.49 ± 0.77 b	29.03 ± 1.75 a
	LMS	11.79 ± 1.05 ab	15.78 ± 0.74 b	20.48 ± 1.31 a	31.83 ± 1.25 a
	LDC	13.23 ± 1.35 a	16.01 ± 0.60 b	19.01 ± 1.06 ab	31.12 ± 1.43 a
	LDS	13.75 ± 1.61 a	17.48 ± 0.99 a	20.83 ± 1.59 a	30.52 ± 1.80 a

Note: different letters indicate significant differences among different treatments (*p* < 0.05). LSD test was used for post hoc analysis. The values represent means ± SE. Sample size: *n* = 5.

**Table 4:** Soluble sugar content of *Indocalamus latifolius* under different treatments.

Ramets	Treatments	Soluble Sugar Content (%)			
		Leaves	Stems	Roots	Rhizome
Mother ramets	UC	6.38 ± 0.33 bc	4.92 ± 0.33 b	3.64 ± 0.14 b	4.46 ± 0.40 bc
	US	6.76 ± 0.21 ab	5.22 ± 0.48 ab	3.69 ± 0.11 b	4.41 ± 0.30 bc
	LMC	6.11 ± 0.22 c	4.77 ± 0.19 b	3.97 ± 0.22 ab	4.37 ± 0.26 bc
	LMS	7.12 ± 0.26 a	5.61 ± 0.32 a	4.14 ± 0.25 a	4.71 ± 0.20 b
	LDC	6.66 ± 0.36 ab	5.01 ± 0.35 ab	3.91 ± 0.23 ab	5.32 ± 0.39 a
	LDS	6.39 ± 0.26 bc	4.75 ± 0.26 b	3.26 ± 0.19 c	4.22 ± 0.14 c
Daughter ramets	UC	6.29 ± 0.28 a	4.61 ± 0.31 ab	3.65 ± 0.21 ab	4.50 ± 0.18 a
	US	6.03 ± 0.25 a	4.55 ± 0.20 ab	3.12 ± 0.17 cd	4.02 ± 0.26 b
	LMC	6.42 ± 0.38 a	4.67 ± 0.27 ab	3.63 ± 0.15 ab	4.66 ± 0.24 a
	LMS	6.00 ± 0.26 a	4.25 ± 0.19 b	2.88 ± 0.20 d	4.04 ± 0.15 b
	LDC	6.23 ± 0.32 a	4.43 ± 0.39 ab	3.94 ± 0.14 a	4.60 ± 0.17 a
	LDS	6.30 ± 0.23 a	4.76 ± 0.26 a	3.32 ± 0.25 bc	4.55 ± 0.25 a

Note: different letters indicate significant differences among different treatments (*p* < 0.05). LSD test was used for post hoc analysis. The values represent means ± SE. Sample size: *n* = 5.

## Data Availability

The data presented in this study are available on request from the corresponding author. The data are not publicly available due to privacy restriction.
